# Photoresponsive molecular tools for emerging applications of light in medicine

**DOI:** 10.1039/d0sc04187d

**Published:** 2020-10-15

**Authors:** Ilse M. Welleman, Mark W. H. Hoorens, Ben L. Feringa, Hendrikus H. Boersma, Wiktor Szymański

**Affiliations:** Department of Radiology, Medical Imaging Center, University Medical Center Groningen Groningen The Netherlands w.szymanski@umcg.nl; Stratingh Institute for Chemistry, University of Groningen Groningen The Netherlands; Departments of Clinical Pharmacy and Pharmacology, Nuclear Medicine and Molecular Imaging, University Medical Center Groningen Groningen The Netherlands

## Abstract

Light-based therapeutic and imaging modalities, which emerge in clinical applications, rely on molecular tools, such as photocleavable protecting groups and photoswitches that respond to photonic stimulus and translate it into a biological effect. However, optimisation of their key parameters (activation wavelength, band separation, fatigue resistance and half-life) is necessary to enable application in the medical field. In this perspective, we describe the applications scenarios that can be envisioned in clinical practice and then we use those scenarios to explain the necessary properties that the photoresponsive tools used to control biological function should possess, highlighted by examples from medical imaging, drug delivery and photopharmacology. We then present how the (photo)chemical parameters are currently being optimized and an outlook is given on pharmacological aspects (toxicity, solubility, and stability) of light-responsive molecules. With these interdisciplinary insights, we aim to inspire the future directions for the development of photocontrolled tools that will empower clinical applications of light.

## Abstract 2

This perspective article explores the current state of light-controlled molecular tools for medical therapy and imaging and offers an outlook on clinical application scenarios and optimisation strategies.

## Graphical abstract

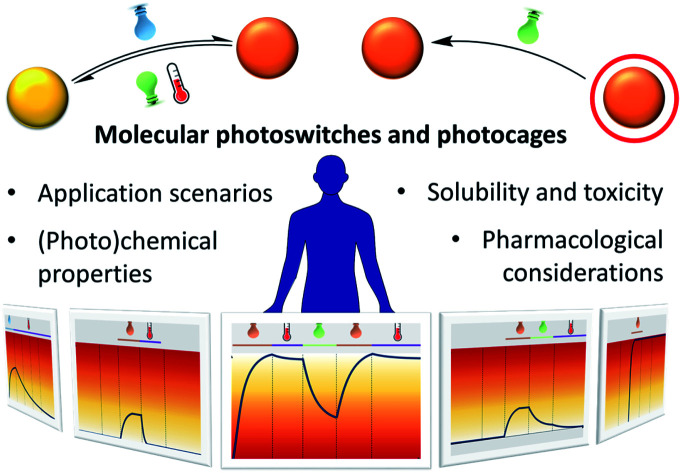

## 1. Introduction

The use of light as a tool for imaging and external control for processes in the human body offers unparalleled bio-orthogonality and spatiotemporal precision.^1^ These advantages have been recognized through the use of light in optical imaging, where light in visible and near-infrared (NIR) regions of the electromagnetic spectrum is used to image the distribution of dedicated tracers in the human body, and in photodynamic therapy, where photosensitizers generate reactive oxygen species (ROS) upon irradiation with light, which is used in cancer treatment. While these applications in therapy and imaging already present a proof-of-principle for the use of light in the hospital setting, we expect that the recent developments in the design and synthesis of photoresponsive molecules will greatly expand the repertoire of clinical modalities, through enabling the use other photochemical processes such as photo-isomerization and photo-cleavage. These light-based approaches will empower drug delivery (nanomedicine), photopharmacology and molecular imaging and will play an important role in modern medicine, allowing for precise and personalized treatment.

In this perspective, we present the recent developments in the field of photoresponsive molecular tools, including photoswitches and photocleavable protecting groups (PPGs), from the perspective of their future medical applications, with special attention to their key (photo)chemical and pharmacological properties. We outline the therapeutic and diagnostic scenarios enabled by those tools and propose how their different properties predispose them to be used in a certain application. Furthermore, we define the key pharmacological aspects that the research on photoresponsive tools should take into consideration in its further development. Finally, we highlight the opportunities and challenges of molecular approaches towards future application of light in medicine, using examples from medical imaging, drug delivery and photopharmacology.

## 2. Photoresponsive molecular tools

**Fig. 1:**
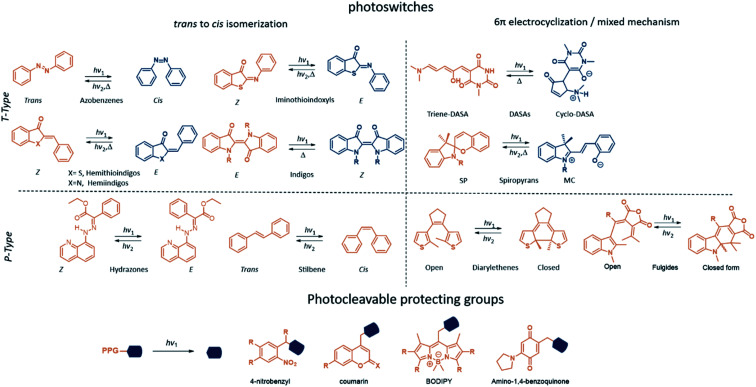
Overview of the photoresponsive molecules for emerging biomedical applications. In blue is shown the metastable state (photoswitches) or the uncaged product (photocleavable protecting groups). In orange is shown the stable state (photoswitches) or the photocleavable protecting groups (PPG).

The molecules discussed here can be categorised in two main classes: photoswitches and photocleavable protecting groups (PPGs) (Fig. 1). What sets them apart is the key photochemical process that they can undergo once promoted to the excited state with light irradiation. While for photoswitches the change in molecular structure is photochemically or thermally reversible, PPGs rely on the irreversible breaking of a σ bond between them and the leaving group of their cargo.

### 2.1 Molecular photoswitches^2–5^

Molecular photoswitches are defined as chemical structures that consists of two or more isomers, that can be interconverted using light irradiation. The photoisomerization of molecular photoswitches proceeds *via* two basic mechanisms: *trans* to *cis* isomerization and 6π electrocyclization of a triene system (Fig. 1). Azobenzenes^6^ and their heteroaromatic analogues,^7^ indigos,^8^ hemithioindigos,^9^ stilbenes,^10^ hydrazones,^11^ and iminothioindoxyls^12^ undergo the double bond isomerisation, while diarylethenes^13^ switch *via* the 6π electrocyclization. Spiropyrans^14^ follow a mixed mechanism, as they first undergo 6π electrocyclization, and later the *trans*–*cis* isomerization takes places. On the other hand, donor–acceptor Stenhouse adducts (DASAs)^15^ and fulgides^16^ first undergo a *trans*–*cis* isomerization, which is followed by a electrocyclization (Fig. 1). Another way to classify photoswitches is to consider the thermal barrier for their re-isomerisation: if the barrier is relatively high, both forms are kinetically stable, and their interconversion is possible only with light (P-type switches). For low barriers, the thermal isomerisation from the metastable (MS) to the stable (S) form will take place (T-type switches). In all cases, the isomerisation results in light-induced modulation of the key properties, such as polarity and geometry. These changes can be used for controlling the behaviour of molecular photoswitches and their derivatives in a biological context.

**Fig. 2:**
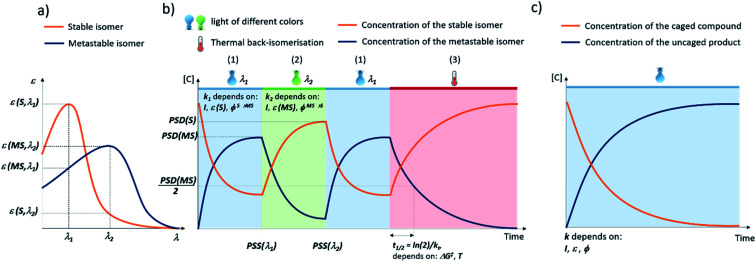
Key properties of light-responsive tools for biomedical applications: molecular photoswitches (a and b) and photocleavable protecting groups (PPGs, c). (a) Idealized UV-Vis spectra of the stable (S) and metastable (MS) isomers of a molecular photoswitch, showing the band separation that enables selective addressing of isomer with light of appropriate wavelengths (*λ*_1_, *λ*_2_). (b) Stages of light-induced isomerisation of a molecular photoswitches. (1) Switching from the stable to metastable state using light of *λ*_1_ wavelength. (2) Switching from the metastable to stable state using light of *λ*_2_ wavelength. (3) In the dark, the system relaxes to the thermal equilibrium. (c) Uncaging of PPG-modified compound is a first-order process, which proceeds with a kinetic constant that depends on the light intensity *I*, molar attenuation coefficient of the PPG at the irradiation wavelength *ε* and quantum yield for the uncaging process *ϕ*.

Fig. 2 presents the key parameters of molecular photoswitches that are relevant to their biomedical applications and that further-on are used to categorize the structures for their applicability in different biomedical scenarios. The idealized UV-Vis spectrum of a molecular photoswitch (Fig. 2a) shows a band separation that enables selective addressing of isomers with light of distinct wavelengths (*λ*_1_ and *λ*_2_). For switching from the stable to metastable state (Fig. 2b(1)) using light of *λ*_1_ wavelength, the kinetic constant *k*_1_ of this process mostly depends on the light intensity *I*, molar attenuation coefficient of the stable form at *λ*_1_ (*ε*(S, *λ*_1_)) and quantum yield for the switching from S to MS *ϕ*^S→MS^. In time, a photostationary state (PSS(*λ*_1_)) is reached, at which the isomerisation in both directions proceeds with the same rate. At PSS, the metastable isomer is enriched, and it is characterised by photostationary state distribution (PSD(MS)) of isomers. Assuming long half-life of the metastable isomer, the content of MS at PSS(MS) depends on the ratio of *ε*(S, *λ*_1_) × *ϕ*^S→MS^ and *ε*(MS, *λ*_1_) × *ϕ*^MS→S^, which represent the efficiency of switching in forward and reverse directions, respectively.

Switching back from the metastable to stable state (Fig. 2b(2)) can be achieved using light of *λ*_2_ wavelength. The kinetic constant *k*_2_ of this process mostly depends on the light intensity *I*, molar attenuation coefficient of the metastable form at *λ*_2_*ε*(MS, *λ*_2_) and quantum yield for the switching from MS to S *ϕ*^MS→S^. In time, a photostationary state (PSS(*λ*_2_)) is reached, in which the stable isomer is enriched, and which is characterised by photostationary state distribution (PSD(S)) of isomers. Assuming long half-life of the metastable isomer, the content of S at PSS(S) depends on (*ε*(MS, *λ*_2_) × *ϕ*^MS→S^)/(*ε*(S, *λ*_2_) × *ϕ*^S→MS^).

In case of the T-type switches, another re-isomerisation pathway is also possible (Fig. 2b(3)). In a thermal process, the system relaxes to the thermodynamic equilibrium, which in most cases consists exclusively of the stable form. This process is characterised by a certain half-life *t*_1/2_ = ln(2)/*k*_t_, where *k*_t_ is the kinetic constant of the thermal MS → S isomerisation, which depends on the free enthalpy of activation associated with this process Δ*G*^‡^ and the temperature *T*.

The ideal properties for a photoswitch (appropriate half-life, photoswitching kinetics, photostationary state distribution of the isomers (PSD) and biological activity) depend heavily on the desired application. However, there are some universal key properties of both photoswitches that are required in biological and biomedical setting. Firstly, they should respond to irradiation with low energy red to near infrared (NIR) light (650–900 nm, so called phototherapeutic window), to enable penetration of light through the skin and into tissue,^1,17^ and avoid the toxicity of carcinogenic UV light.^18,19^ Secondly, photoswitches should also be nontoxic, biologically stable and resistant against photodegradation (fatigue). Furthermore, building a photoswitch into *e.g.* a pharmacologically active substance should not induce its toxicity or dramatically change its original properties. These last four general key parameters are discussed in depth later (Section 7).

### 2.2 Photocleavable protecting groups (PPGs)^20^

The application of a PPG relies on transiently blocking the function of a compound, which is restored after irradiation with light through irreversible bond cleavage. The PPGs discussed in this review are of the following types: 4-nitrobenzyl (*o*NB) and coumarin derivatives, BODIPY-type PPGs and amino-1,4-benzoquinone-based systems (Fig. 1).

Deprotection of a PPG-modified compound is a first-order process, which proceeds with a kinetic constant that depends on the light intensity *I*, molar attenuation coefficient of the PPG at the irradiation wavelength *ε* and quantum yield for the uncaging process *ϕ* (Fig. 2c). The product of *ε* and *ϕ* is known as the uncaging cross section and is a good measure of the efficiency of the process, as it takes into account both the probability of molecule entering the excited state and the probability of photocleavage taking place. High uncaging cross section means that light of lower intensity is sufficient for activation, which is a key concern in strongly attenuating media such as human tissue.

Additional requirements for the use of PPGs in medical applications include the following: (i) activation wavelength in the phototherapeutic window, (ii) clean deprotection to biologically benign products^20^ (iii) solubility and stability in aqueous solutions.

## 3. Scenarios for application of photocontrolled tools in medicine

**Fig. 3:**
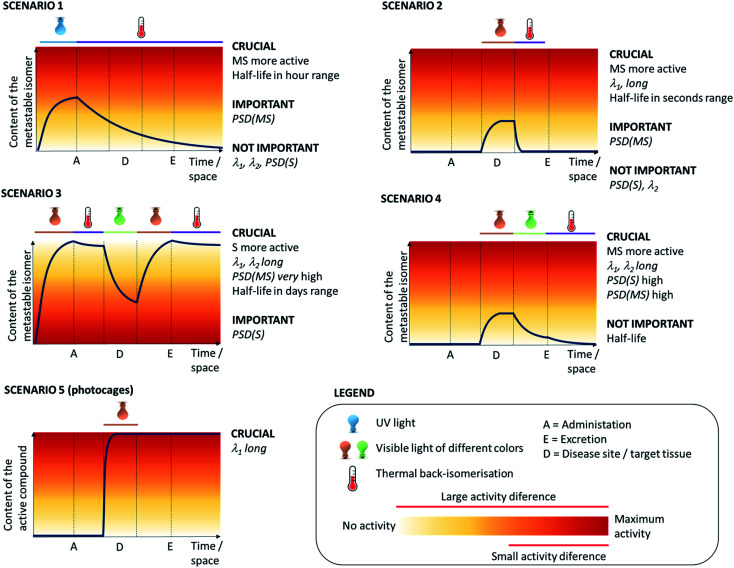
Therapeutic scenarios, for the use of reversibly (1–4) and irreversibly (5) photocontrolled systems for medical application, concerning the situations when the compound is to be activated outside (1) or inside (2–5) of the body, and taking into consideration if the stable (3) or metastable (1, 2 and 4) form of the switch is more active in the biological system. The intensity of colour in the background of the graphs represents the increasing biological activity that can be explored with changing the concentration of the metastable isomer (blue line). See Section 2.1 in the text for the explanation of the abbreviations used.

The specific application of the photoresponsive molecule dictates the requirements regarding activation wavelength, photostationary state distributions, photochemical conversion efficiency and the half-life of the metastable state (Fig. 2). However, it is important to note that there is not one privileged combination of those parameters, and depending on the application, certain scenarios can be defined (Fig. 3) that rely upon different photoswitch properties. In the following, we outline those scenarios and then discuss the optimisation of each key parameter discussed separately with reported examples.

### 3.1 Scenario 1

In this scenario (Fig. 3), the photoresponsive molecule is activated before the administration to the human body. Once administered, it slowly loses its activity through thermal relaxation and is then excreted in its inactive state.

The prerequisites for this scenario are the MS state being more active in the biological system and having the half-life in the hour range, to make sure the photoresponsive molecule is still active upon arrival at the desired site. Since the irradiation happens outside the body before drug administration, wavelengths outside of the phototherapeutic window can be used for activation. A key parameter in this scenario, similarly to scenarios 2–4, is the activity difference between the two photoisomers. Idealized case (Fig. 3) assumes a large difference, therefore the photostationary state distribution (PSD(MS)) for the active form does not necessarily need to be high to enable a pronounced difference in biological response. However, more realistically, this high difference in activity between the isomers is difficult to achieve. Here, the PSD of the metastable active form should be high to still have impact. Photoresponsive molecules that fulfil these requirements are most of the T-type photoswitches with half-lives in the range of hours, including certain types of azobenzenes,^6^ hemithioindigos^9^ and spiropyrans.^14^

**Fig. 4:**
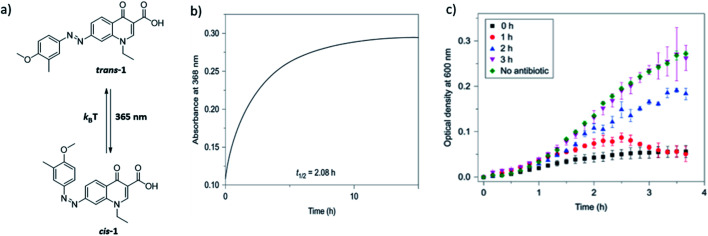
Azobenzene-derived photoswitchable antibiotic **1**. (a) Compound *trans*-**1** is a weakly potent antibiotic, which can be isomerized to its more active *cis*-**1** form using UV irradiation. (b) The *cis* form has a half-life of ∼2 h, and isomerizes back to the *trans* form, in a first order process characterised by the recovery of the absorbance band at 368 nm. (c) Freshly activated antibiotic **1** prevents the bacterial growth, as indicated by the lack of increase of optical density in bacterial culture in time. Increasing the time between activation and addition to bacterial culture (0–3 h) results in partial thermal re-isomerisation to the less active *trans*-**1** isomer and is accompanied by the drop in potency to inhibit bacterial growth. After 3 h, the sample loses its potency and behaves the same as the negative control. Reproduced from ref. 21 with permission from Springer Nature, Copyright 2013.

Scenario 1 is useful in cases where one aims at limiting the exposure of the environment to the biologically active substance. This is especially important in *e.g.* antibiotic therapy, where the presence of active antimicrobial agents in soil and fresh water has been shown to lead to emergence and spread of bacterial resistance. This has driven the development of photoswitchable antibiotics,^21^ in the design of which the criteria of the metastable form being more potent and having hour-long half-lives have been taken into account. Velema *et al.* envisioned an example of this scenario, where the quinolone-based azobenzene derivative was activated with 365 nm to the *cis* form (Fig. 4, compound **1**). The antibacterial activity was reduced when the active *cis* form reverted thermally to the less potent *trans* form with a half-life of ∼2 hours.^21^

### 3.2 Scenario 2

In this scenario (Fig. 3), a photoresponsive molecule is inactive in its thermally stable (S) state, in which is administered to the human body and switched to the active form at the target site. Outside of the irradiated volume, it switches spontaneously back to the inactive state in a fast, thermal process. This scenario allows for the highest level of control over the active photo-isomer compared to other scenarios, and thus represents a preferred situation for numerous applications.

**Fig. 5:**
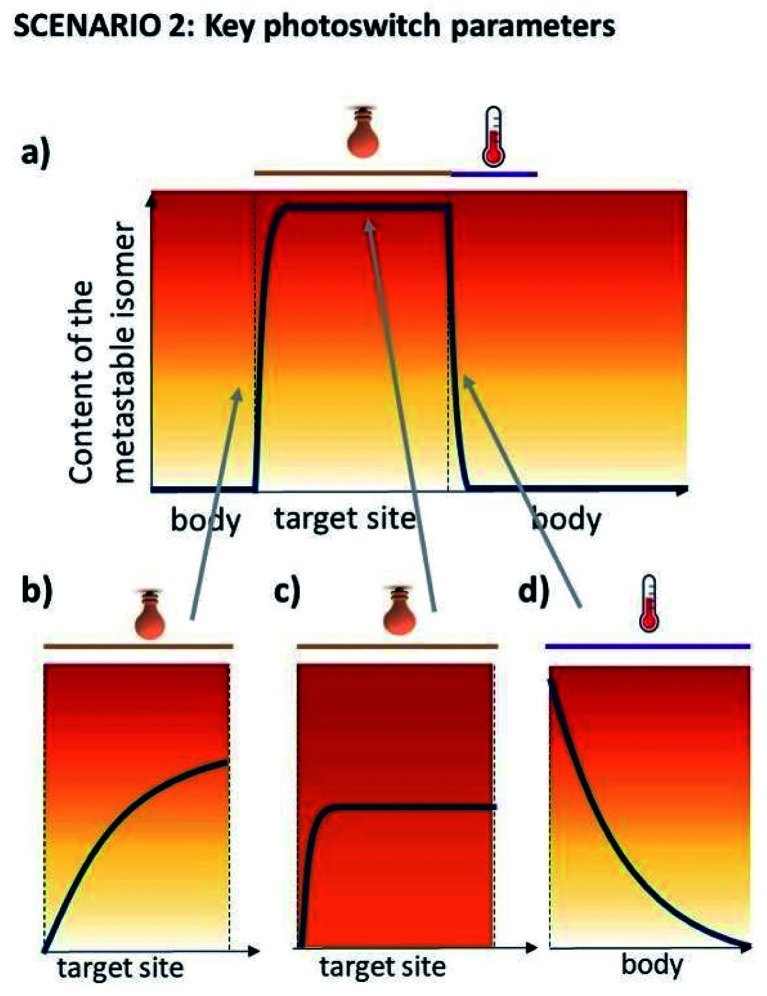
Key photoswitch parameters for scenario 2. (a) Light- and temperature-induced changes in the content of the metastable, biologically active isomer (ideal situation). In panels (b–d), consequences of insufficient photoswitching cross section (panel b), PSD(MS) (panel c) and thermal relaxation rate (panel d) are shown.

The key aspect for this scenario is the short half-life, which is used to inactivate the molecule outside of its intended action site, rendering the PSD of the thermally stable state (PSD(S)) unimportant. Furthermore, light is used for switching *in vivo* in one direction, meaning that only one wavelength used for operation has to be in the red-NIR range. However, especially when there is no high fold difference in activity between the two forms, the PSD of the metastable state should be high to ensure the biological impact. Fig. 5 presents the consequences of sub-optimal photoswitch performance for scenario 2 (Fig. 5a). Insufficient forward photoswitching cross section (Fig. 5b) may lead to limited exposure of the target to activated compound or even prevent reaching the PSD, as the forward switching competes with fast thermal re-isomerisation. Low PSD(MS) (Fig. 5c) may result in limited difference of biological effect evoked by irradiation, especially when the difference in potency of photo-isomers is small. Finally, slow thermal re-isomerisation (Fig. 5d) leads to the unnecessary or even harmful exposure of the body to the active form.

Photoswitches that could fulfil all those requirements are still scarce. Azonium species derived from protonation of tetra-*ortho*-methoxy-azobenzenes respond to red-NIR irradiation and show half lives in the micro secondo range,^22,23^ making them compounds-of-choice for this application. DASAs show absorbance in the 450–700 nm region and half-lives in the sec-min regime, but their function is compromised in aqueous environment.^15^*N*,*N*-Disubstituted indigos can be isomerised at 500–670 nm, and re-isomerize on the sec-min timescale, but again their water solubility is a limitation.^24^ Finally, the recently reported iminothioindoxyls show millisecond-range half-lives and stability/solubility in aqueous media, but their absorption bands reach only to the green range of the UV-Vis spectrum.^12^

**Fig. 6:**
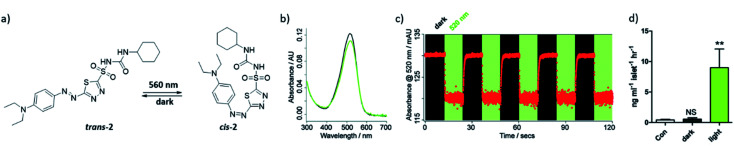
(a) Azosulfonylurea **2** used for the control of pancreatic beta cell function. (b) The UV-spectrum of compound **2** in the dark (black line) and under irradiation with 520 nm light (green line) (c) cycles of photoswitching between *cis*- and *trans*-**2**, with in green irradiation with 520 nm and in black under dark conditions. (d) Rodent islets treated with compound **2**, under dark or under light (560 nm) conditions, the response is increasing of insulin secretion. No difference was obtained between dark conditions and the control (Con, 5 mM glucose-alone). Reproduced from ref. 25 with permission from The Royal Society of Chemistry, Copyright 2015.

Scenario 2 is useful in cases where short half-life is sufficient and highly localized action is warranted, for example in triggering the neuronal response by regulating the action of ion channels. Broichhagen *et al.* showed an example of an azobenzene for the control of pancreatic beta cell function (Fig. 6, compound **2**). Upon irradiation with 560 nm light, the inactive *trans* isomer is switched to the active *cis* form, which quickly relaxes back to the *trans* form (half-life is in a millisecond time range).^25^

### 3.3 Scenario 3

While the cases, in which the metastable (MS) isomer is the more active one, are preferred (see scenario 2), the azologisation of flat, biologically active molecules often results in a light-controlled drug in which the stable (S) photo-isomer is more potent.^26^ In those situation, scenario 3 (Fig. 3) is particularly useful, in which the photoresponsive molecule is switched off by irradiation (*λ*_1_) before administration to the human body. After it is reaching the target site, it is switched back (*λ*_2_) to its active form. Around the target site, the photoresponsive molecule is switched back (*λ*_1_) to its inactive form, to prevent non-selective action while it is being excreted from the body.

Besides the higher activity of the stable form, the other requirement for this scenario is the long half-life (or even the use of a P-type switch), which is needed to ensure that the photoresponsive molecule does not switch back to the active form, which would lead to undesired side effects. Furthermore, the activation wavelengths required for the both the activation and inactivation should ideally be in the red/NIR light range, to enable operations inside the human body. Also, the PSD(MS) should be very high, to enable efficient switching-off of the activity prior to administration. Again, the scenario shown in Fig. 3 represents an ideal situation where the PSD(S) is of lesser importance. In more realistic case, however, the difference between the two forms is limited, and it is important to achieve a high PSD of the thermally stable form to observe a difference in the biological system being regulated.

Photoswitches that could fulfil all those requirements are P-type diarylethenes and fulgimides,^27^ which show absorbance in the 450–700 nm region, but the geometrical changes that can be obtained upon isomerization are limited, which renders the design of diarylethene-containing photocontrolled tools for biomedical application challenging.^13,28^ Tetra-substituted azobenzenes could also be a possible candidate for this scenario, as they show absorbance in the green light region of the spectrum and half-lives in the days regime.^29,30^

**Fig. 7:**
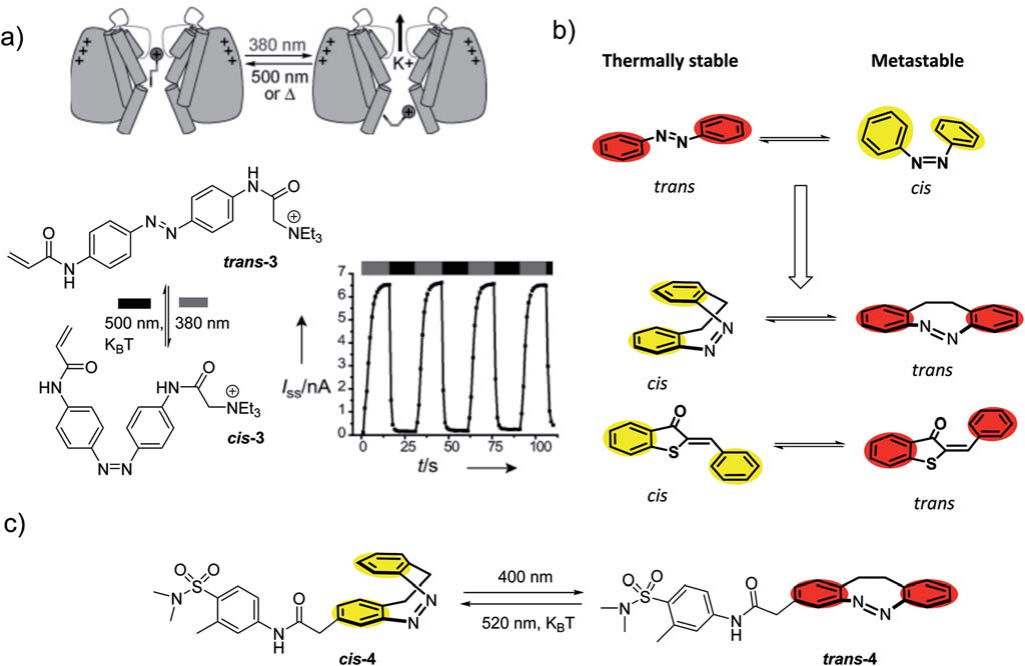
(a) Photoswitchable blocking of voltage-gated potassium channels by compound **3**, which shows higher potency in the *trans* state, as an example of compounds useful in scenario 3. Reproduced from ref. 31 with permission from Wiley-VCH, Copyright 2009. (b) “Sign inversion” approach used when the more potent photoisomer (in red) is showing higher thermal stability. (c) The potassium channel blocker/opener diazocine **4** designed through “sign inversion” approach by Trads *et al.*^33^

Although this scenario is not ideal, due to the needed deactivation before administration, it is often the only option in cases where the stable isomer shows a higher activity. An example has been presented by the group of Trauner,^31^ who used molecule **3** (Fig. 7a) as a photoswitchable blocker of the voltage-gated potassium channel. While *trans*-**3** was the more potent isomer, thanks to the considerable thermal half-life of the *cis* isomer and the high (>80%) PSD(MS) that can be reached under *λ* = 380 nm, it was possible to generate this form first, and then use light of *λ* = 500 nm to switch it to the more portent form. While this example does not yet provide the possibility to use red light for switching in both directions, it efficiently shows the level of control (Fig. 7a) that can be achieved using scenario 3.

Furthermore, an alternative approach was recently developed for azobenzene-based agents that show higher activity in their stable form. This strategy, dubbed “sign inversion”, relies on other photoswitches, such as hemithioindigos^32^ and diazocines^33,34^ (cyclic azobenzenes) (Fig. 7b). Especially the latter offer an elegant alternative to linear azobenzenes, since their *cis* form, whose geometry defines the biological activity, is the thermally stable one. This is due to the fact that most active azobenzenes are in the elongated stable form (the *trans* isomer), while in diazocines the thermally stable-isomer (*cis*) is bent.^33,34^ An illustrative example was shown by Trads *et al.* (Fig. 7c), who used functionalized diazocines as potassium channel blockers/openers.^33^ The design was based on prior observation that classical azobenzene-modified molecules showed higher potency in the *trans* form (see compound **3**). The use of diazocine (compound **4**) resulted in the *trans* form still being more potent, but in this case thermodynamically less stable, enabling transient photoactivation of the stable *cis* form upon irradiation with 400 nm light.^33^

### 3.4 Scenario 4

In this scenario, the photoresponsive molecule is administered in its inactive, thermally stable form. At the target site, it is activated by irradiation at *λ*_1_, while irradiation of the surrounding tissues at *λ*_2_ results in the decrease of activity outside the intended site of action.

**Fig. 8:**
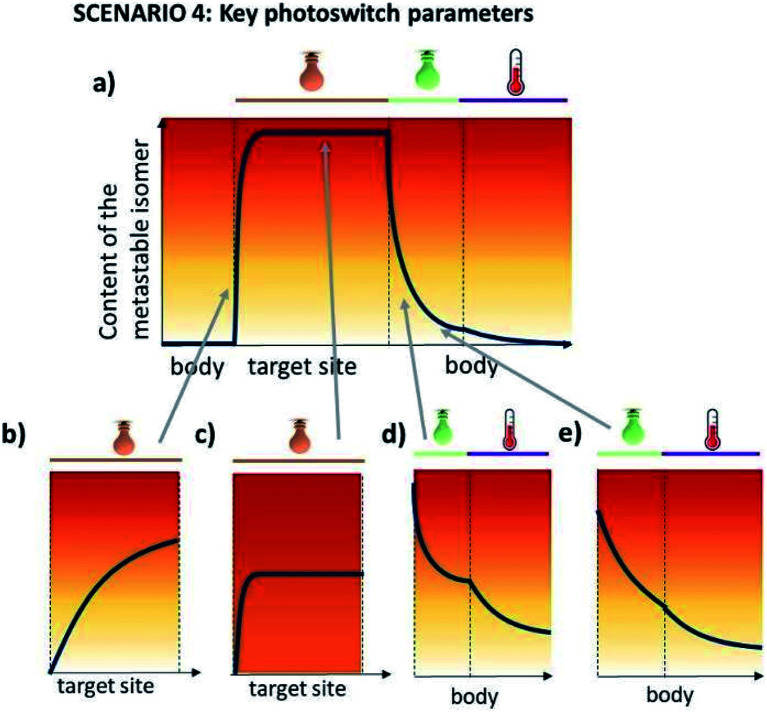
Key photoswitch parameters for scenario 4. (a) Light- and temperature-induced changes in the content of the metastable, biologically active isomer (ideal situation). In panels (b–e), consequences of insufficient forward photoswitching cross section (panel b), PSD(MS) (panel c), PSD(S) (panel d) and insufficient backward photoswitching cross section (panel e) are shown.

In this case, the metastable form is the one that shows higher activity, however, its long half-life is not a limitation, in fact it can help in achieving a high PSD(MS). The key requirement in scenario 4, which severely limits the repertoire of photoswitches suitable for it, is for both of the isomers to be addressable in the red/NIR light. Fig. 8 further elaborates on the critical properties of photoswitches used in scenario 4 (Fig. 8a). Insufficient forward (Fig. 8b) and backward (Fig. 8e) switching cross sections cause the insufficient exposure of target and harmful exposure of the body to the active compound, respectively. Low PSD(MS) (Fig. 8c) may result in limited biological effect increase under irradiation, especially when the difference in potency of photo-isomers is small. Finally, low PSD(S) leads to the exposure of the body to the active form.

Photoswitches that fulfil these requirements are scarce, since the necessity for the both isomers to absorb in the red/NIR region of the spectrum with good band separation poses a challenge hitherto unmet. Promising systems include indigo switches,^8^ diarylethenes,^13^ tetra-*ortho*-methoxy-substituted azobenzenes^29^ and azonium compounds^35^ as long as the photoswitch can be fully or almost fully switched to its less potent state.

**Fig. 9:**
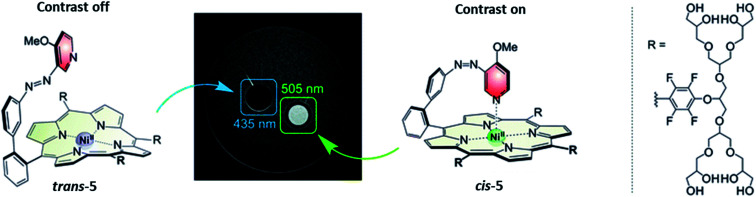
The photoresponsive MRI contrast agent **5**. In the *trans* isomer shows no MRI contrast, while upon isomerization with *λ* = 505 nm to the *cis* isomer the contrast is switched on. This process can be reversed by irradiation with *λ* = 435 nm light. Reproduced from ref. 37 with permission from PCCP Owner Societies, Copyright 2019.

This is an ideal scenario, where the photoresponsive molecule upon administration does not show any activity and can be activated with spatial and temporal control with light. An example of this was reported by Herges and co-workers (Fig. 9). The photoresponsive MRI contrast agent (compound **5**) is active in the *cis* form (MS), resulting in faster magnetic relaxation of surrounding protons, while when the photoresponsive contrast agent is in the *trans* form, no MRI contrast is observed. The contrast could be switched on, upon irradiation with 505 nm light, and needs to be switched off with 435 nm light due to the long half-life of 39 days (in water at 37 °C). Although this example does not yet fulfil all the requirements of this scenario, as the activation wavelength in the red/NIR light is still lacking, it shows a promising starting point for further optimisation.^36,37^

### 3.5 Scenario 5

In the final scenario (Fig. 3), the photoresponsive molecule is activated after administration to the human body. However, in this case, the activation is irreversible – either due to the nature of photoresponsive agent (PPG *vs.* photoswitch) or due to the process being triggered (*e.g.* an irreversible cargo release).

Again, an important parameter is the activation wavelength, since the activation is envisioned to be inside the human body. Other key points include (i) the stability of the caged prodrug, because premature release can lead to undesired side effects and (ii) the uncaging efficiency, since aside from being responsive to red light activation, the photoresponsive unit should feature high quantum yield of the uncaging process for this process should not require long irradiation times.^20,38^

**Fig. 10:**
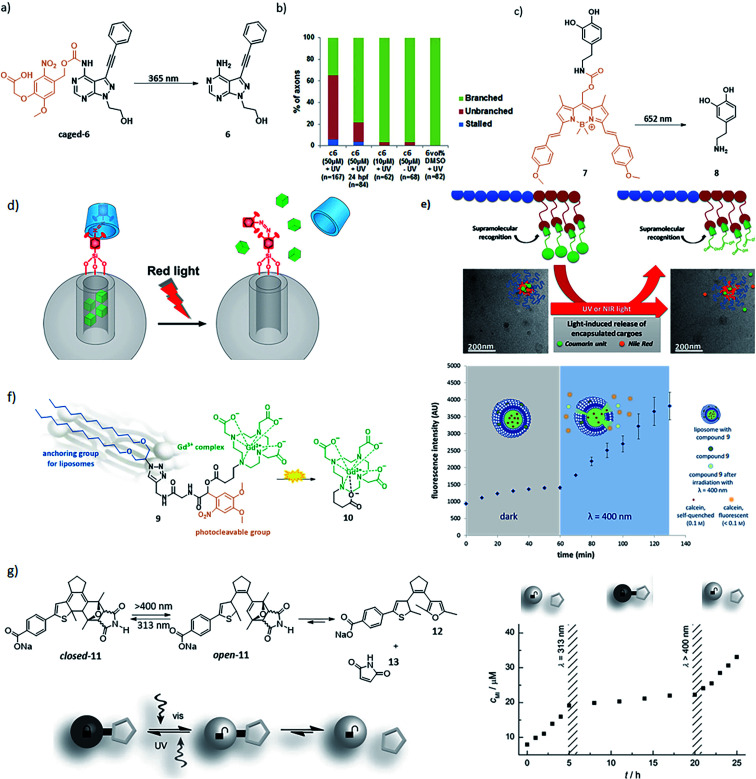
Examples of application of scenario 5 in drug activation, delivery and medical imaging. (a) PPG-protected proto-oncogene tyrosine-protein kinase (RET) inhibitor **6**, which prevents motoneuron extension and axonal pathfinding during development in zebrafish. (b) Quantification of axonal phenotypes in the different treatments with compound c**6** (caged-**6**) and **6** in different concentrations and with different post fertilization (hpf) incubation times (*n* = number of axonal processes quantified). Reproduced from ref. 39 with permission from Springer Nature, Copyright 2015. (c) BODIPY-protected dopamine derivative activation for controlling heart rhythm. (d) Light-responsive supramolecular valves for photocontrolled drug release from mesoporous nanoparticles. Reproduced from ref. 42 with permission from the American Chemical Society, Copyright 2016. (e) Supramolecular block copolymers as novel UV and NIR responsive nanocarriers based on a photolabile coumarin unit. Reproduced with permission from ref. 44 from Elsevier, Copyright 2020. (f) A light-responsive liposomal agent for MRI contrast enhancement and monitoring of cargo delivery, showing the increase of fluorescence intensity upon light-induced release of model cargo. Reproduced from ref. 45 with permission from The Royal Chemical Society, Copyright 2019. (g) Left: furyl-substituted DAE undergoes retro-Diels–Alder upon ring opening under irradiation with *λ* = 400 nm light, resulting in a release gradual of compound **13**. Right: the gradual release rate of maleimide (**13**) can be reversibly activated by switching compound **11** to its open form with *λ* = 400 nm light and back to the closed form with *λ* = 313 nm light. Reproduced from ref. 43 with permission from Wiley-VCH, Copyright 2015.

This relatively simple, yet powerful approach has been used to enable local drug activation (Fig. 10a and c) and release (Fig. 10d–g). An important application for this scenario is the use of photocaged (PPG-modified) bioactive compounds, where the part of the compound, which is crucial for its biological activity, is blocked with a PPG and upon photocleavage the compound becomes active (Fig. 10a and b). An example of this was reported by Bliman *et al.*, where a PPG was incorporated into a proto-oncogene tyrosine-protein kinase (RET) inhibitor (compound **6**).^39^ RET is involved in the development and maintenance of neurons of the central and peripheral nervous system. The authors protected the amine functionality, and upon irradiation with *λ* = 365 nm light, the inhibitor **6** was released showing a 12-fold increase in potency. The uncaging of the inhibitor was also performed *in vivo* (zebrafish embryos) and was shown to effect motoneuron development (Fig. 10b). Sitkowska *et al.* recently showed how low energy red light can be used for controlling biological activity, by reporting a BODIPY-protected dopamine derivative (Fig. 10c, compound **7**). The light depended release of compound **8** was tested in spontaneously beating hESC-derived cardiomyocytes, upon irradiation with 652 nm light for 1 min, there was an increase in heart beat frequency observed.^40^

Applications of scenario 5 in drug delivery^41^ can be achieved by constructing drug carriers that feature light responsive release systems (Fig. 10d and g), or whose structure integrity is responsive to light (Fig. 10e). The example of the first approach (Fig. 10d) employs of red-light-responsive tetra-*ortho*-methoxy-azobenzene photoswitches, which form supramolecular interactions with β-cyclodextrins to create a cap on the silica nanoparticles loaded with cargo. Red-light-induced *trans*–*cis* isomerisation of the switch results in the loss of interactions with the cap and therefore in the cargo release.^42^ The concept of using photoswitches for uncaging on molecular level was further exploited by Göstl and Hecht who designed a furyl-substituted diarylethene **11** (Fig. 10g). Upon irradiation with *λ* = 400 nm light, compound **11** switches from its closed to open form, which enables the release of compound **12** and maleimide **13** in a retro-Diels–Alder reaction. The retro-Diels–Alder reaction could be reversibly switched off again by closing the diarylethene back to its unreactive form.^43^

Another drug carrier approach (Fig. 10e) is represented by the use of amphiphilic block copolymers that featured a light responsive, coumarin-based PPG incorporated into the side chain. The block copolymers were able to self-assemble into micelles of about 25 nm diameter when dispersed in water, trapping the cargo. Upon irradiation with 365 nm light, the micelles disassembled. This effect could also be achieved under irradiation with NIR light (730 nm), using two-photon absorption phenomenon, albeit with much lower efficiency (Fig. 10e).^44^ Finally, the use of scenario 5 in medical imaging-guided drug delivery was reported by Reeβing *et al.* Here, a Gd^3+^-based MRI contrast agent **10** was linked *via* a PPG to lipophilic alkyl chain (resulting in compound **9**), which could dock into DOPC-based liposomes (Fig. 10f). These liposomes could be filled with cargo, and upon irradiation with 400 nm light the cargo could be released, with the simultaneous change in the size of the contrast agent, which could be followed by MR imaging to prove efficient photoactivation.^45^ The examples above illustrate the applications of scenario 5, however they do not yet fulfil all the requirements stated above, especially with respect to red light activation and/or high uncaging efficiency. However, they represent a promising set of proofs-of-principle that will inspire further applications of this relatively most simple, yet powerful approach.

## 4. Shifting the photoresponsive molecule activation wavelength to the red/NIR range

In all the scenarios described above that rely on the local activation in human body, the wavelength of light used for this purpose is of crucial importance, since light needs to penetrate deep through the skin and into tissues in order to reach the photoresponsive molecules. The preferred activation wavelength is between 650–900 nm (red light to near infrared light).^18,19,46^ Although most of the photoresponsive molecules used today are activated with UV light (see also the examples above), in recent years a considerable amount of research has been performed to bathochromically shift the absorption bands. In the following sections, we summarise the strategies that are taken towards shifting the activation wavelength to red/NIR light. The approaches taken in enabling the visible light activation fall generally into three categories: (i) changing the band gap in the photochromic unit itself, (ii) taking advantage of two-photon absorption processed, and (iii) using indirect photoexcitation through energy of electron transfer.^47^ Since the first approach is the most specific to the photochromic tools, we discuss it below in more detail.

**Fig. 11:**
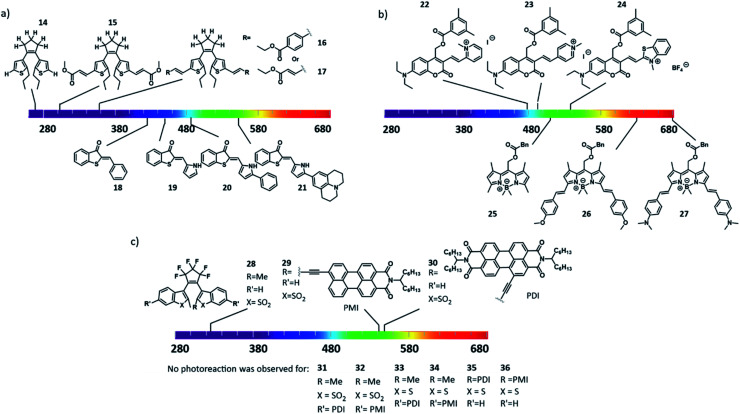
Extension of the π-conjugation as a method to red-shift the activation wavelength of molecular photoswitches (a and c) and photocages (b).

The first strategy taken to enable direct excitation at higher wavelengths is the increase of π-conjugation in the chromophore, which leads to the increase in HOMO and decrease in LUMO energy levels. This general approach is applicable to all chromophores. Tosic *et al.* have reported its use for diarylethenes, which were modified increase the conjugation (Fig. 11a, compounds **14–17**), which lead to bathochromically shift of the absorption band (from 300 nm to 420 nm).^48^ Zweig *et al.* reported different π-extended pyrrole hemithioindigo photoswitches (Fig. 11a, compounds **18–21**), where the stilbene core was replaced by a pyrrole, which increased already the activation wavelength, while arylation of the pyrrole increased it even further.^49,50^

The increase in conjugation can also be applied to shift the activation wavelength of various PPGs, as was shown by Bojtár *et al.*, who reported coumarins containing a heterocycle moiety which included a quaternary nitrogen atom (Fig. 11b, compounds **22–24**). Besides increasing the activation wavelength, the charge also increase the solubility in aqueous solvents.^51^ Another example of the use of increase π-conjugation was reported by Peterson *et al.*, who presented BODIPY-type PPGs that were conjugated with styryl groups (Fig. 11b, compounds **25–27**), allowing operation above 700 nm.^52^

However, increasing the π-conjugation is not always efficient, as was shown by Irie and co-workers, who aimed to bathochromically shift the absorption spectra of diarylethenes. While promising results were obtained when increasing the π-conjugation with a perylenemonoimide (PMI) and perylenediimide (PDI) dye on the R position (Fig. 11c, compound **29** and **30**), when introducing the same substituents at the R′ position at the aromatic ring (compounds **31–36**), the diarylethene lost its photoswitch ability.^53^ Increasing the π-conjugation can also be applied on azobenzenes, where it results in shifting the π–π* transition band, commonly used to evoke the *trans*–*cis* isomerisation.^47^ However, this can result in overlap with the lower energy n–π* band, which is used to induce the reverse *cis*–*trans* isomerization, making it harder to switch the isomers selectively.^47^ Altogether, increasing the conjugation is a general approach that often provides the desired effects. However, it also commonly results in the decrease of aqueous solubility and has thus limited application in biomedical context.

**Fig. 12:**
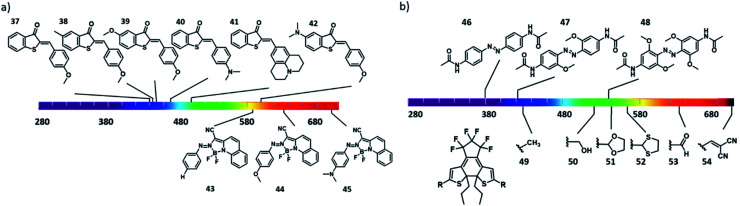
Introduction of electron withdrawing and donating substituents in conjugated positions in photoswitches to create a “push–pull” system and extend the absorption spectra towards the red/NIR region in (a) hemithioindigos and azo-BF_2_ switches; (b) azobenzenes and diarylethenes.

Another strategy used to enable direct excitation with light of lower energy is to take advantage of the so-called push–pull substitution pattern, in which a strongly electron donating and withdrawing molecules are placed in conjugation on the chromophore. While often used for azobenzene-based dyes, it is also efficient in increasing the activation wavelength of hemithioindigo photoswitches. Here, the thioindigo fragment can be seen as the acceptor moiety, while the stilbene acts as the donor part. This effect can be enhanced by increasing the stilbene donor capacity through substitution.^9^ However, this can lead to decrease of thermal stability of the *trans*-isomer.^54^ Kink *et al.* reported an increase activation wavelength by increasing the donating effect by introducing a substituent on the *para* position of the stilbene group (Fig. 12a, compounds **37–42**)^55^, and upon protonation of the amine this donating effect can be switched off.

On one hand, using push–pull systems is a viable option for applications in aqueous environment, because the resulting chromophores show highly pronounced solvatochromism and the band-shifting effect is stronger in polar media.^47,56^ On the other hand, however, it also commonly results in a decrease of the thermal stability of the metastable form of the photoswitchable molecule. This strategy is therefore particularly useful in scenario 2, where short half-lives and red-shifted activation wavelengths are crucial, as shown by Trauner and co-workers in the photocontrol of pancreatic beta cell function with molecule compound **3** (Fig. 6).^25^ Compound **2** could be switch with 560 nm to the active *cis* form (compound **2**, with a half-live in sub-second range), which binds to Epac2A, allowing optical control of cell function and insulin secretion. Another approach that is used to engineer the position of the bands is through complexation using electron pairs in the chromophore. Aprahamian and co-workers reported azobenzenes with a complexation of the azo group to BF_2_, which lead to bathochromic shift the π–π* band. Further optimization of the R group (Fig. 12a, compounds **43–45**) enabled the isomerisation with red light of 730 nm.^57^

Finally, a strategy that has proven especially useful for designing visible light responsive azobenzenes in recent years relies on the use of lower energy transitions – namely the absorption bands associated with the symmetry-forbidden n–π* transitions. In normal azobenzenes, those bands have low intensity and are poorly resolved for both isomers, and are therefore used only for reverse *cis*–*trans* switching, taking advantage of the usually higher quantum yield for this transition over the forward one. However, increasing the n–π* band separation, which enables selective addressing of the photoisomers, has enabled many successful designs that are already used in biomedical context. The first use of this strategy dates back to the discovery of the photochemistry of diazocines by Herges *et al.*, where the band separation was achieved through geometrical constrain, which also lead to inverse thermal stability^58^ (also explained in Section 3.3). Later, Woolley and co-workers reported a tetra-*ortho*-methoxy substituent pattern for azobenzenes to separate the n–π* transitions by bathochromically shifting the band of the *trans* isomer (Fig. 12b, compounds **46–48**).^59^ The authors hypothesized that this was due to the twisting of the *trans*-isomer and due to the interaction of the methoxy groups with the lone pair of the nitrogen of the azo group. Further increase of the electron density on the chromophore rendered it basic enough to undergo protonation at physiological pH, and enabled activation with NIR light.^23^ A similar bathochromically shift of the absorption bands was reported for the tetra-*ortho*-chloro and tetra-*ortho*-alkylthio substituted azobenzenes.^29^ A complementary strategy was taken by Hecht, Bléger and co-workers, who hypsochromically shifted the n–π* absorption band of the *cis* isomer to achieve better band separation in a tetra-*ortho*-fluoro substituted azobenzenes, also resulting in a very high thermal stability of the metastable form.^60^

Finally, the influence of substituents on the bathochromic shift of the absorption bands can me more subtle. For ITI photoswitch, introducing an electron donating substituents on the benzene ring at the *para* position of the nitrogen atom increases the absorption wavelength. This effect is due to the change in the twist around the =N–C– single bond: the electron donating substituents increase the electron density of the phenyl ring, which leads to an increase in the conjugation with the thioindoxyl moiety.^12^ Placing substituents next to the sulphur atom on diarylethenes also has effect on the activation wavelength. Pu *et al.* reported diarylethenes baring different substituents next to the sulphur (Fig. 12b, compounds **49–54**). The authors showed that electron-withdrawing substituents increase the activation wavelength and also increase their cyclization quantum yield, while electron donating substituents increased the molar absorption coefficients.^61^

In summary, it needs to be stated that although the above mention strategies are promising, it is a daunting task to increase the activation wavelength of the photocontrolled molecular tools without severely affecting their other key properties (absorption band separation, half-life of the metastable form and pharmacological properties) and each of the new designs needs to be re-evaluated taking into account all these aspects.

## 5. Improving photostationary state distributions for molecular photoswitches

Obtaining a large difference in biological activity between isomers is not readily achieved, and incomplete photo-isomerization to either the stable or metastable photo-isomer adds a photochemical challenge to acquire large differences in biological activity. As described in Fig. 2, PSD in the forward switching direction is defined by the ratio of *ε*(S, *λ*_1_) × *ϕ*^S→MS^ and *ε*(MS, *λ*_1_) × *ϕ*^MS→S^ and therefore is influenced by the absorption band overlap.

Most of the known photoswitches cannot be quantitatively switched between the two forms using light, which presents a major challenge to the efforts on increasing the activation wavelength (see Section 4 and discussions on band separation therein). However, over the years some strategies have been reported to increase band separation and help optimize the PSD achievable for switching in both directions.

**Fig. 13:**
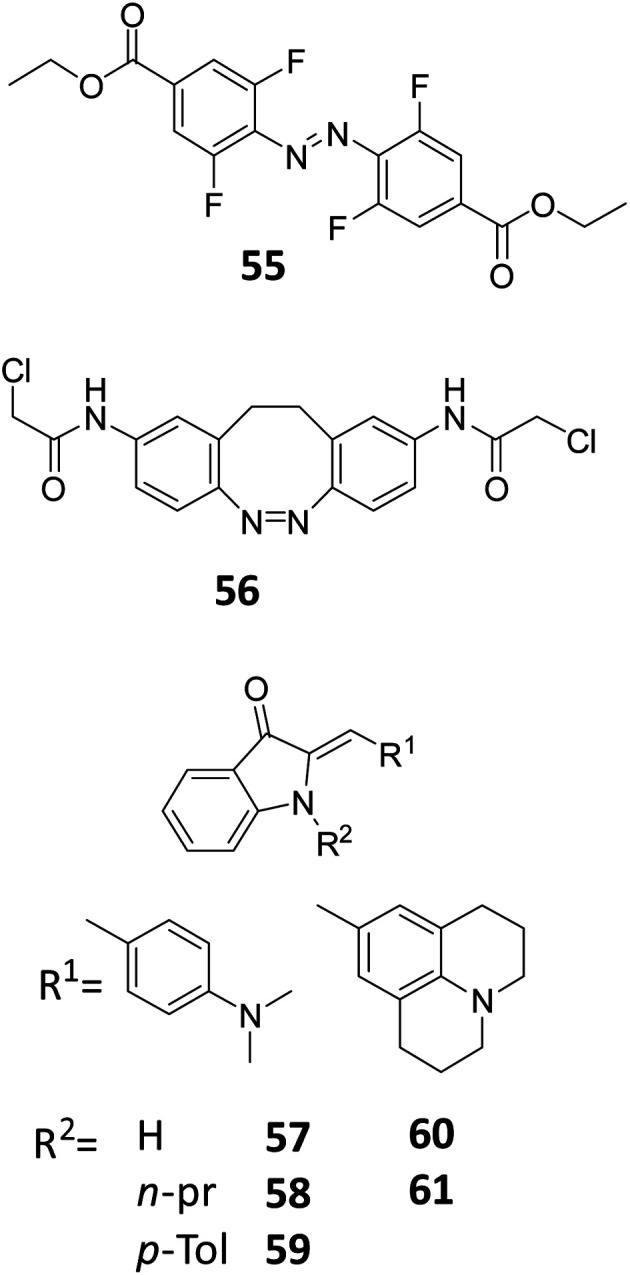
Overview of photoresponsive molecules discussed in the engineering of photostationary state distributions (PSD).

For classical, UV-responsive azobenzenes, an empirically established^62,63^ design principle is the introduction of alkoxy-substituents in the *para* position, resulting in >90% efficiency of switching in both directions due to the efficient separation of π–π* bands. For the visible-light responsive systems that rely on n–π* transitions, the aforementioned use of *ortho*-substituted azobenzenes, especially the fluorine substituted ones (Fig. 13, compound **55**), results in high PSD ratio (90% *cis* under 500 nm irradiation, 97% *trans* under 410 nm irradiation).^60^ Furthermore, diazocines are known to feature large separation between the *cis* and *trans* n–π* transitions. For an example molecule **56**, used by Woolley and co-workers to photocontrol peptide conformation, the authors reported to be able to almost fully (99.7%) isomerize it back to the thermally stable *cis* form with 518 nm light.^64^

Petermayer *et al.* reported that, while increasing the activation wavelength by increasing the push pull system of the hemiindigo (compounds **57–61**, Fig. 13) with amine substituents on the stilbene moiety, as well as placing substituents in the nitrogen from the indigo part, they also obtained high PSD and long half-lives (in the day to year range). For compound **61**, it was shown that upon irradiation with 470 to 505 nm, more than 95% of the *trans* isomer was formed, while with irradiation with 680 nm, 99% was converted back to the *cis* isomer. The high PSD and half-lives are not compromised if the nature of the solvent is changed (from toluene to THF to DMSO). These properties are due to the conformational differences of the *cis* and *trans* isomers, where full planarity and conjugation of the chromophore can be achieved only in the *trans* isomer.^65^

## 6. Engineering the half-life of the metastable state

The stability of the metastable state is the final parameter that defines the suitability of molecular photoswitches for different scenarios Fig. 3. While both short and long-living species have their applications, it is important to be able to understand the factors that influence the thermal stability to be able to engineer it to one's needs.

**Fig. 14:**
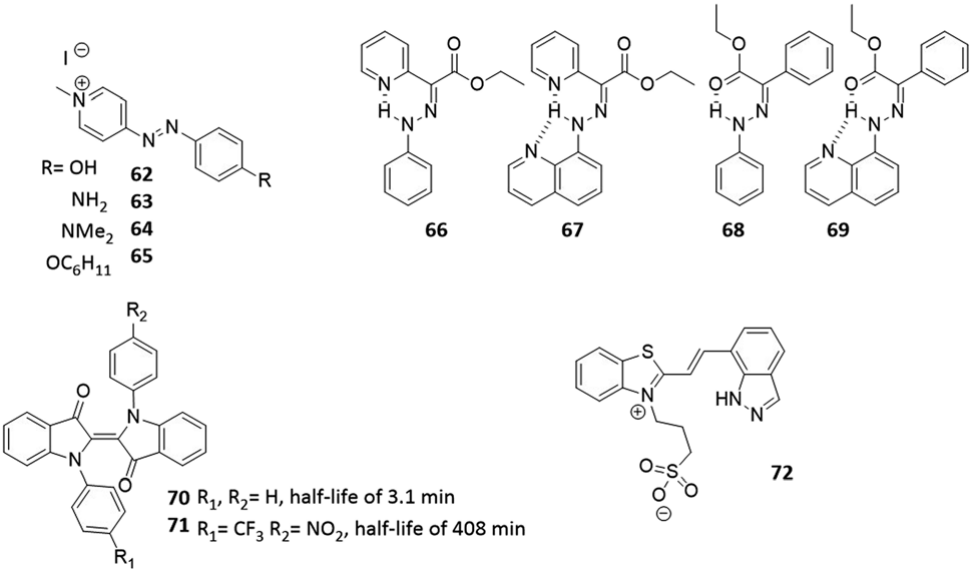
Overview of the photoresponsive molecules to discussed for engineering the half-life.

For azobenzenes and heteroaryl azo dyes, the influence of substituent on the half-life are the best understood,^66^ which enables the adjustment of this property from nanosecond, in extreme cases of push–pull systems (see molecules **62–65**),^67^ to years.^60^ The latter has been achieved in case of tetra-*ortho*-fluoro substituted azobenzenes, which, besides enabling visible-light activation, also lead to a higher stability of the *cis* isomer, due to the stabilization of its n orbital, while the n orbital of the transition state is less stabilized, which leads to a higher barrier for the *cis* to *trans* isomerization. Knie *et al.* demonstrated this for compound (Fig. 13, compound **55**), which showed half-life of two years in DMSO.^60^ Another photoswitch that is known to possess extremely high thermal stability of the metastable isomer is the hydrazone structure (Fig. 14, compounds **66–69**), reported by the group of Aprahamian.^11^ Those molecules feature half-lives in the centuries timescale and can be considered to be bistable for all practical purposes.

As discussed in Section 4, the push–pull strategy applied to HTI can negatively influence the half-life and PSD of the HTI, due to a decrease in thermal stability of the metastable isomer. However, this can be prevented by placing electron-donating groups on the *para* position of the sulphur atom, which stabilize this form. An example of this was shown by Kink *et al.*, where an amine moiety was placed *para* to the sulphur (Fig. 12, compound **42**), the obtained HTI showed increased half-life (up to 30 days) and enabled reaching the PSD for each isomer of was around 80%.^55,68^ Hecht and co-workers described an approach to increase the half-life of *N*,*N*-disubstituted indigos (Fig. 14, compounds **70** and **71**), an emerging class of promising photoswitches that can be activated by red light, but still suffer from stability issues in aqueous media. By incorporating electron withdrawing substituents on the aromatic rings (Fig. 14, compound **71**), the thermal stability of the metastable *cis*-isomer could be increased (half-life from 3.1 min to 408 min), while the switch could still be operated with 660 nm light and its stability in water was improved.^24^ Recent studies also showed an improvement on the half-lives of spiropyrans. Abeyrathna *et al.* reported that by incorporating an indazole ring instead of the phenol (Fig. 14, compound **72**), the PSS and also the half-life (to 48 h) were both enhanced.^69^

Altogether, the recognition of the importance of half-life as the key parameter that defined the application area of photocontrolled tools has inspired many recent reports on controlling this property. While it is still intertwined on a molecular level with absorption band separation and position, rendering the selective optimisation of half-life without affecting the *λ*_max_ and PSS a major challenge, continued research on structural fine tuning supported by calculations might enable this in the future.

## 7. Pharmacological properties of photocontrolled molecular tools

At the current early stage of the development of photoresponsive molecules for applications in medicine, the focus often lies on maximising the difference in behaviour between the irradiated and non-irradiated molecules (see Section 3). The recognition of the importance of the colour of light for the penetration depth has also driven the development of new molecular tools (see Section 4). Those two factors represent of course *condiciones sine quibus non* for future applications. However, pharmacological factors,^70^ such as stability in water and cellular environment, polarity and toxicity are also key parameters that will have to be addressed in the future before the light-controlled molecules can make it to the bedside. Furthermore, they can positively inspire new approaches, where for example two photoswitches that operate in unison are applied:^71^ one for controlling the interaction with the molecular target (pharmacodynamics), and one for controlling the pharmacokinetic properties of the photoresponsive construct, to influence its ADME profile. In the following sections, we summarise the recent efforts towards studying and optimising these parameters for PPGs and molecular photoswitches.

### 7.1 Stability in water and in the cellular environment

**Fig. 15:**
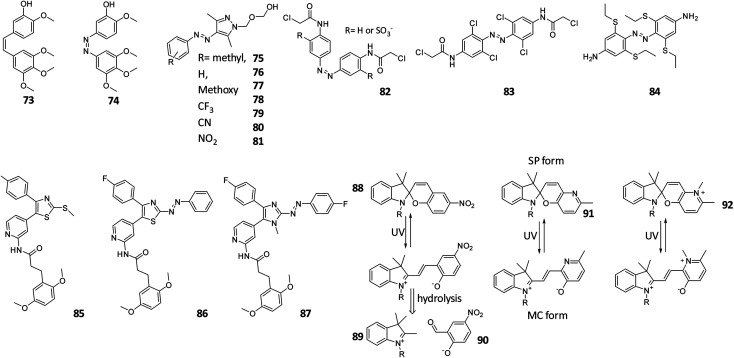
Overview of discussed photoswitches for the stability in water and in the cellular environment.

In general, our understanding of the aqueous and metabolic stability of molecular photoswitches and PPGs is only being developed in recent years. An exception to this are the azobenzene photochromes, which have been used as dyes in food and clothing industries for many years.^72^ This has resulted in comprehensive knowledge of their behaviour and interactions with biomolecules. Azobenzenes are degraded through reduction with glutathione (GSH) or *via* enzymatic metabolism.^19,29,73^ Since the resulting anilines are toxic,^74^ special care needs to be taken to test for the susceptibility of designed azobenzene photoswitches towards GSH reduction.^73,75,76^ For example, compound **74** (Fig. 15) is a derivative of combretastatin A-4 (compound **73**), which is a colchicine domain MT inhibitor, whose isomerisation from its *trans* to *cis* form is accompanied with impressive and rarely observed 250× increase in potency, as reported first by Borowiak *et al.*,^77^ followed by and Engdahl *et al.*^78^ However, further investigation by Sheldon *et al.*^79^ revealed that compound **74** was prone to GSH degradation and that the light activated *cis*-isomer degraded faster than the *trans*-isomer.^77–79^

The susceptibility of azobenzenes to reduction by GSH depends strongly on their substitution pattern. Since the reaction is initiated by a nucleophilic addition of the GSH thiol group to the diazo moiety,^80^ increasing the electron density on this bond lowers the reduction rate. Several examples illustrate this effect. Stricker *et al.* introduced an electron rich pyrazole as one of the aromatic rings in a diazo molecule. The resulting arylazopyrazoles were stable when incubated with GSH for 22 h (Fig. 15, compound **75–81**).^75^ Woolley and co-workers showed that a *para* substituted electron rich azobenzene **82** (Fig. 15), which was used as fluorescent reporter in zebrafish embryos showed no significant changes in the UV-Vis spectrum or in the thermal relaxation rate after the incubation of the azobenzene with GSH for 24 h.^81^

However, a balance needs to be found when increasing the electron density to prevent GSH reduction, since beyond a certain point it will render the diazo bond Brønsted-basic enough to be protonated at physiological pH, again increasing the sensitivity towards degradation.^19,29,73^ This was observed by Woolley and co-workers,^73^ who synthesized multiple tetra-*ortho*-substituted azobenzenes with the aim to bathochromically shift the n–π* absorption band of the *trans* isomer (Fig. 12, compound **48** and Fig. 15, compound **83**). And observed that the azobenzene with the more electron-donating methoxy substituent was degraded upon incubation with GSH. The authors concluded that the tetra-*ortho*-methoxy azobenzene was more easily protonated, which increases the electrophilicity of the azo bond. A later publication by the same authors showed how to prevent the degradation while still maintaining beneficial photochemical properties: after changing the methoxy substituents to alkylthio substituent (Fig. 15, compound **84**), the azobenzenes proved to be stable against GSH, while the wavelength absorption was still increased.^73,76^

Importantly, it is not only GSH that can reduce the diazo bond in biological assays. A degradation of the azobenzene through a different pathway was reported by Scher *et al.*, who studied 2-aza-thiazole-based compounds **85–87** as photoswitchable inhibitors of kinases p38αMAP kinase and casein kinase 1δ (CK1δ). In the *in vitro* kinase assay, compound **86** showed higher activity in its *cis*-form, which did not correspond to the prediction from the *in silico* docking studies. After further investigation, supported by crystal structures of the switches bound to proteins, the authors identified the reduced hydrazine form of compound **86** as the potent inhibitor. However, in this particular example, dithiothreitol (DTT), which is commonly added to prevent oxidation of the protein in the kinase assays, was responsible for the reduction. Since *cis*-isomer is more prone to reduction than the *trans*-isomer, the active hydrazine was present in higher concentration in the irradiated sample, explaining the observed higher potency.^82^ Again, using a more electron rich, *N*-methyl-imidazole-based analogue **87** prevented the reduction, highlighting the feasibility of this approach.

As opposed to azobenzenes, other molecular photoswitches and PPGs have been much less studied with respect to their stability in biological context. However, it is known that spiropyrans in their merocyanine (MC) form are prone to hydrolysis, since water can easily attack the ene-iminium cation, which is followed by a retro-aldol reaction (Fig. 15, compound **88–90**), resulting in degradation, as was reported for compound **88**. To reduce this problem, over the last few years spiropyrans have been developed that are more stable against the hydrolysis.^83–85^ An example has been presented by Heckel and co-workers, who reported that the hydrolysis can be almost entirely prevented by replacing the nitro-phenol ring by pyridine (compound **91**) or *N*-methyl pyridinium (compound **92**) ring (Fig. 15).^83,85^ The increased stability against hydrolysis is most likely due to the fact that the thermal equilibrium lies to the spiropyran side.

**Fig. 16:**
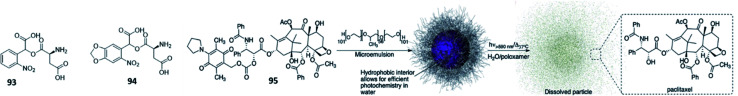
Overview of discussed PPGs for the stability in water and in the cellular environment. For compound **95**, the accompanying figure is reproduced from ref. 87 with permission from The Royal Society of Chemistry, Copyright 2016.

The importance of stability under physiological conditions has also been recognized for PPGs. Since their stable attachment to the caged molecules in the dark is the prerequisite for successful application, their optimisation has also been focussed on this aspect. For example, in an effort to improve stability for *ortho*-nitrobenzyl (*o*NB) protecting groups, Zaitsev-Doyle *et al*. found that the methylenedioxy substituted PPG **94** was more stable towards hydrolysis then compound **93**.^86^ A completely different approach to improve stability was shown by Carling *et al.* paclitaxel, dexamethasone, and chlorambucil where caged with amino-1,4-benzoquinone. However, since this protecting group is not stable in aqueous media, the authors incorporated the unstable photocaged compound **95** in water-dispersible nanoparticles. This way, the amino-1,4-benzoquinone PPG was protected by the hydrophobic core of the nanoparticles from being degraded (Fig. 16). Upon light irradiation with 590 nm, the caged compound was released.^87^

Altogether, stability in biological context poses and important design criterion for photoresponsive tools, which has recently stimulated research addressing this particular issue. It is important to test the stability in early stages to be able to make design changes to prevent this.

### 7.2 Toxicity

Before photoresponsive molecules can enter clinical trials and be used in medical applications, it is necessary to perform *in vivo* toxicity studies.^88^ The extent of these toxicity studies will depend on the target amount to be injected. *E.g.* for microdosing studies in humans, the initial toxicity tests may be limited. Doses above the microdosing range of 100 μg will require more elaborate investigations.^89^

However, since the majority of the designed photoresponsive molecules fails to reach that state, the general knowledge on their toxicity is low. Importantly, sometimes the toxicity coincides with the desired bioactivity, as is the case in chemotherapy. This is being taken advantage of in the field of photoactivated chemotherapy (PACT),^90^ where both the PPG, which here is a metal complex, and the caged chemotherapeutic become toxic upon activation. However, since in most cases the photoresponsive element itself if required to be non-toxic, we focus here on general undesired toxicity of photocontrolled tools.

Because azobenzenes have been used for various purposes, including food dyes, for decades, most information about their toxicity is available.^72,74^ While, as a class of compounds, they do not show general toxicity, azobenzenes are known to be degraded by enzymatic metabolism. Thus great care has to be taken to prevent degradation *via* bacterial azoreductases or by intestinal microflora, which break the azobenzenes down to anilines. It is therefore important to test not only the azobenzene for toxicity but also their corresponding metabolites.^72,74^ Furthermore, as signalled before, it would be beneficial to develop molecules with a second photoswitch that regulates the metabolism after being pharmacologically active to prevent toxic reactions and improve efficient removal from the body.

Chung and Cerniglia reported that the anilines that result from azobenzene degradation can be further metabolized to genotoxic compounds. They studied the structures of many mutagenic azo dyes and found that compounds containing *p*-phenylenediamine and benzidine moieties where especially mutagenic. They also reported that for *p*-phenylenediamine the toxicity can be decreased by sulfonation, carboxylation, deamination, or *via* substitution of one of the hydrogen of the amino group by ethoxy or an acetyl group. For the benzidine moiety, it was found that complexation with copper ions or formation of salts decreases the mutagenicity.^91^

**Fig. 17:**
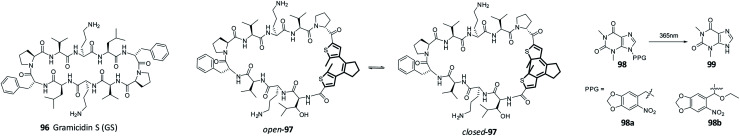
Overview of the photoresponsive molecules discussed in the context of toxicity.

Recently, Babii *et al.* published a toxicity study of diarylethene photoswitches (Fig. 17). The diarylethene was incorporated into a cyclic peptide gramicidin S (**96**), which has been designed for exhibiting anti-cancer properties.^92^ The authors observed a difference in the cytotoxicity *in vitro* between the closed and open form of compound **97**. However, when the same study was performed *in vivo*, the difference in cytotoxicity between the open and close form was reduced. It was hypothesized that this is due to the fact that the closed form has less favoured pharmacokinetics. After administration, the closed form was detected in kidney, liver and blood plasma in high concentrations. The authors also investigated phototoxicity of the photoswitchable drug. They saw that half of animals kept in the dark showed signs of toxicity up to 2 hours after treatment, while the animals in daylight showed toxicity signs at least till 6 hours after the treatment. These findings highlight the importance of performing *in vivo* toxicity tests.

In the design of PPGs, toxicity plays an important part because the removed cage should not show general toxicity.^18,93^ It is known that *o*NP-based PPGs can form harmful by-products upon photocleavage (Fig. 17, compounds **98** and **99**).^18^ An example for preventing the formation of those by-products was shown by Young and Deiters.^94^ Different PPGs blocking the 9-NH position of theophylline where tested. By changing the PPG to (2-nitro-valery)oxymethyl (NVOM) (Fig. 17, compound **98b**), the authors prevent the formation of toxic benzaldehyde. Instead, acetophenone is released upon uncaging, which is known to be more benign. Due to the high risk of toxicity, it is important to test it for the photoresponsive molecules as soon as possible after the proof-of-concept for an application is shown. Often, it is preferred to perform such test also in animal models or organoids, as preliminary test in single cells cultures do not adequately model metabolism in humans.^95,96^

### 7.3 Solubility

A balanced solubility is a key parameter in the development of effective bio-active molecules. Considerations that play a role in this is whether compounds should be able to pass membranes, whether therapeutically relevant concentrations can be achieved and how fast compounds are cleared from the patient.

Frequently solubility is characterized by the log *P* value, which describes the partition coefficient of solubility between a solution at physiological pH and 1-octanol. In addition to this model, aqueous solubility can also be described as the equilibrium between a compound in solution and it its most stable crystal form, hence melting temperature has been a predictor for solubility. Combining both, the General Solubility Equation (GSE) describes:^97^log *S*_w_ = −0.01(*M*_p_ − 25) − log *P*_octanolwater_ + 0.5,where *S*_w_ is molar aqueous solubility of an organic non-electrolyte in water and *M*_p_ is the melting point (in degrees Celsius).

Low aqueous solubility of small molecule drugs has been a challenge for the field of medically chemistry for decades and generally two strategies can be derived from the GSE. First, one can improve aqueous solubility by chemically modifying drug with solubilizing groups, such as by increasing the number of hydrogen bond donors and acceptors or adding groups with soft or hard charges. Secondly, one can decrease the melting point of a compound, for example by removing aromaticity or otherwise decreasing the flatness of the compound.^98,99^ We recognize that many light-responsive tools are large flat aromatic molecules with poor aqueous solubility, which severely limits their medical use. Besides solubility reasons, extension of compound lipophilicity also bear the risk of accumulation in fatty tissues, and thus enhances the potential for long term toxicity of the substance in humans.^100^ Here, we discuss several examples on strategies to improve aqueous solubility of light-controlled tools and how these approaches affect other properties.

**Fig. 18:**
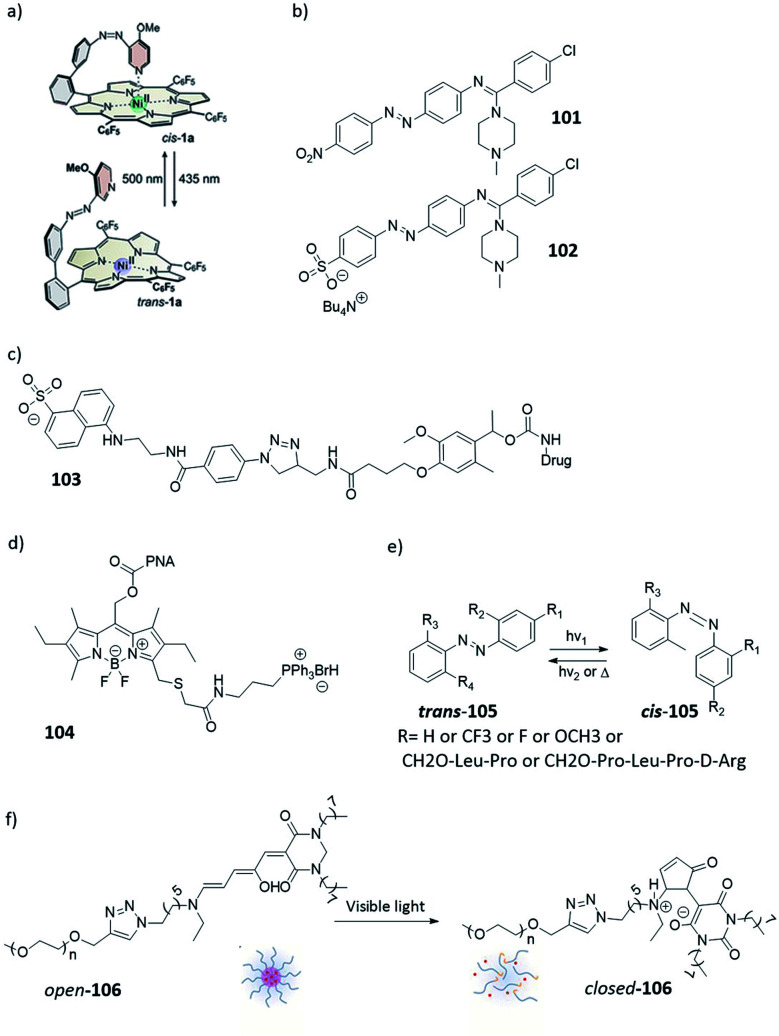
Overview of photoresponsive molecules discussed in the context of controlling aqueous solubility. (a) Introduction of PEG chains to improve solubility; reprinted from ref. 36 with permission from American Chemical Society, Copyright 2015. (b) Introduction of a solubilizing group to improve solubility. (c–d) Controlling biological distribution; (e) Modulating biological activity through solubility. (f) Drug release through light-induced increase in polarity; reprinted from ref. 102 with permission from American Chemical Society, Copyright 2014.

A common strategy to increase aqueous solubility is the addition of polyethylene (PEG) chains. This approach was reported by Dommaschk *et al.* to improve the solubility of the first generation photo-switchable contrast agents for MRI (see Fig. 18, compound **100**), where the contrast agents was switched off when the azobenzene moiety was in the *trans*-isomer due to change in coordination number of the nickel(ii).^36^ However this design had solubility issues and all measurements where performed in DMSO.^36^ In a later reported, next generation photoswitchable contrast agent, the solubility issue was solved by linking glycerol dendrimers to the porphyrin complex (Fig. 9). Although the solubility was increased, the switching was less effective and for some complexes it was found that the nickel ion was lost from the complex.^36,37^ This example perfectly demonstrates that any modification of the original molecule may cause functional changes hampering essential properties. Moreover, one needs to be aware on the potential impact of such changes on *e.g.* biodistribution of the new compound. In radiopharmacy, these effects can be shown quite easy, whereas they may remain unseen during the clinical development of a photoswitch containing compound. It may even be beneficial to perform biodistribution experiments using for instance ^14^C or ^11^C labelled-molecules, in order to be able to evaluate the pharmacokinetics and biodistribution of a novel compound.^101^ By doing this, it can be predicted how suitable the particular compound will perform in animals and humans.

In general, light-responsive molecules are based on rather flat, aromatic, lipophilic systems. Furthermore, red-shifting of their absorption bands, while necessary for their application (see Section 4), often relies on increasing the conjugation and leads to even less water-soluble, flat aromatic molecules.^83^ An example of this challenge, and an elegant solution, was shown by Rustler *et al*., who studied photoswitchable antagonists for the histamine H_1_ receptor. The authors achieved the bathochromic shift of the absorption band by using a push pull azobenzene design (Fig. 18, compounds **101** and **102**). However, this resulted in reduced solubility, which was recovered upon the introduction of a sulfonate group on the aromatic ring (compound **102**).^103^ Further examples showing the effectiveness of adding sulfonate groups for solubility were provided by the groups of Woolley^104^ and Feringa.^105^

Gratifyingly, it is not always the case that when the activation wavelength is increased it results in solubility issues. Bojtár *et al.* showed that, while trying to increase the activation wavelength of the coumarin PPG by extending the conjugation with a cationic, *N*-alkylated pyridine moiety, they also achieved the improved the solubility (Fig. 11, compounds **22–24**).^51^ However, careful consideration is required when adding charged functional group(s) to the photoresponsive molecule to render them more soluble, as this can influence their ability to cross biological membranes, which is crucial for altering the activity of intracellular drug targets. A drug containing a negative charge such as a sulfonate does not allow for efficient diffusion passed the cell membrane, while permanently positive charges such as *N*-alkylated pyridines tend to accumulate in mitochondria. Those effects are of course not necessarily a limitation. Example of turning this effect to an advantage was reported by Dcona *et al.* (Fig. 18, compound **103**), who modified a drug with a sulfonate moiety, attached through a photocleavable linker. Due to the presence of a negative charge, this molecule was not able to enter the cells. However, upon irradiation with light (*λ* = 365 nm), the anticancer drug was released from the linker and crossed the cell membrane.^106^ In principle, this strategy can be applied to any cell permeable drug molecule as long as it has reactive groups to which the light responsive linker can be attached.^106^ Another example that uses charges to regulate the compound biological distribution dynamically with light was reported by Kand *et al.* By adding charged functional groups on the BODIPY-based PPG, they could achieve the selective targeting to cell organelles (Fig. 18, compound **104**). The authors showed that by adding triphenylphosphonium to the BODIPY high accumulation was found in the mitochondria.^107^

Besides structural changes to help improve the solubility, another important point to keep in mind during the design of a photoswitchable bioactive system is that both isomers need to be soluble in aqueous solutions to prevent aggregation,^108^ which can lead to results that are difficult to interpret and highly depend on the type of medium, its ionic strength, co-solvents added, *etc.* However, change in solubility can also be taken advantage of. This was shown by Brown *et al.*, who studied different azobenzenes (Fig. 18, compound **105**) and observed different solubilities for the *trans* and *cis*-isomer, the *cis*-isomer was in most of the cases more soluble in aqueous media. This effect can shift the *cis*–*trans* equilibrium in favour of the *cis*-isomer.^109^

Another way to turn the change in solubility to an advantage was shown with DASA photoswitches that, upon photoswitching, turn form planar, linear, hydrophobic form to a cyclic, zwitterionic one. Helmy *et al.* incorporated a monomethyl PEG chain into the DASA photoswitch through azide–alkyne cycloaddition reaction. In aqueous environment, the resulting molecules of compound **106** self-assembled into micelles. The micelles could be disassembled through cyclisation induced with yellow light (*λ* = 570 nm), which changed the polarity of the molecule (Fig. 18f).^102^

## 8. Optimisation of photocontrolled tools: towards applications or towards assays?

In their development, the activity of biologically active molecules is tested on isolated enzymes,^110^ cells, *ex vivo* tissues^111^ or model organisms,^112^ long before a molecule ever might enter clinical trials. The high demand for predictive pre-clinical models to evaluate biological activity has resulted in a large toolbox of biological assays. Yet, we recognize that many of these tests have been developed prior to the time when light controlled tools emerged and therefore might not be fully compatible with testing light-controlled biologically active compounds. This leads to molecular design dilemmas signalled in the title of this section: should we optimise the tools towards their final application (see clinical scenarios in Section 3), or towards the assays that we use to determine their biological activity? Working towards the real application seems to be the obvious answer, but it cannot proceed without first showing the activity and its photomodulation *in vitro*.

An obvious example of this dilemma is presented by engineering the half-life of the metastable state. For example, many cell-based assays rely on overnight incubation, which is achievable for compounds with long thermal half-lives. Assaying long-living photo-isomers is experimentally much easier then compounds with short thermal half-lives, even though we recognize that short half-lives are a desired feature in achieving high spatial resolution to empower final applications.

Another challenge comes from the fact that the orthogonality of light in biology has also been recognized in the development of biological assays, long before the advent of photopharmacology and related techniques. Many such tests rely on measuring absorbance^63^ (*e.g.* many cell viability assays), fluorescence (most enzyme activity assays) or luminescence (cellular assays using reporters^111^). Obviously, using such assays to measure activity of compounds that themselves are photoactive and photoresponsive poses problems. While solutions are actively being sought after from the perspective of photocontrolled tools (*cf.* the establishing of GFP-orthogonal microtubule inhibitors by Thorn-Seshold and co-workers),^113^ it is clear that also new assaying modalities are required that would provide short experiments without the necessity of optical input or readout. In that respect the use of electrophysiological^114^ and radiometric assays^115^ seems advantageous and should be further pursued in the future.

## 9. Conclusion

Over the years great progress has been made towards the design of photoresponsive molecules with the longer-wavelength or improved half-life, PSD and the pharmacological properties. Despite these achievements, there are still challenges that remain to be addressed before the translation to the clinic of these highly interesting compounds can take place.

Importantly, the broad spectrum of potential applications, as highlighted here by the scenarios defined in Section 3, renders it impossible to give one set of desired properties for a photocontrolled tool. We hope that the considerations presented here will inspire the development and optimisation of new molecular photoswitches and PPGs towards different applications, and we are delighted to observe the significant expansion of the light-responsive molecule toolbox in recent years. While appreciating all the effort put into optimising their (photo)chemical properties, we also invite the respective communities to consider the pharmacological aspects of photoswitches and PPGs, to ultimately enable bringing light to the clinics taking advantage of molecular level precision.

## Conflicts of interest

There are no conflicts to declare.
